# South African flag sign

**DOI:** 10.1093/omcr/omad104

**Published:** 2023-10-23

**Authors:** José Martín Alanís Naranjo, Julio César Rivera Hermosillo

**Affiliations:** Department of Cardiology, Hospital Regional 1° de Octubre ISSSTE, Mexico City, Mexico; Department of Cardiology, Hospital Regional 1° de Octubre ISSSTE, Mexico City, Mexico

A 80-year-old woman with type 2 diabetes and hypertension presented to the emergency department with acute chest pain. High-sensitivity Troponin I level was 9045 ng/ml (reference value, <19 ng/ml). The electrocardiogram (ECG) demonstrated a complete right bundle branch block as well as ST-segment elevation in leads I, aVL, and V2 with ST-depression in lead III (Fig. 1A), aligned with the South African Flag (Fig. 1B). Transthoracic echocardiography showed only anterior wall hypokinesia and a LVEF of 56%. Emergency coronary angiography revealed occlusion of the first diagonal (D1) vessel and thrombus with TIMI grade 2 flow (Fig. 1C); stent placement was not possible as the vessel was <2 mm in diameter, so an intravenous antiplatelet treatment was administered. The patient’s clinical course was uneventful, and she was discharged symptom-free with medical treatment for secondary prevention.

**Figure 1:**
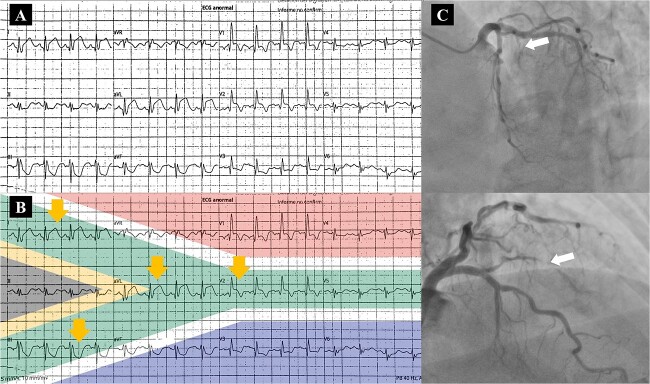
High lateral myocardial infarction. (**A**) ECG with ST-segment elevation in leads I, aVL, and V2 with ST-depression in leads III and aVF. (**B**) Flag of South Africa superimposed on ECG, showing the characteristic pattern (arrows). (**C**) Coronary angiography with occlusion of the first diagonal vessel and thrombus with TIMI grade 2 flow (arrows).

A high lateral myocardial infarction (HLMI) can often be missed due to noncontiguous ST elevation on the electrocardiogram (ECG) [1]. The pattern of ST-segment elevation in lead I, aVL, and V2 with ST-segment depression in lead III is considered a sign of acute occlusion of the D1 branch of the left anterior descending coronary or HLMI, also known as the South African Flag sign [1–3], where the pattern follows the green stripe of this flag, like our case (Fig. 1B). The D1 occlusion results in a projection of the ST-segment vector toward I, aVL, and V2 and away from III and aVF [1]. Identifying ischemic ECG changes early is crucial for timely revascularization and limiting left ventricular dysfunction.

## CONFLICT OF INTEREST STATEMENT

None declared.

## FUNDING

None.

## ETHICAL APPROVAL

Not applicable.

## CONSENT

Written consent from the patient was obtained prior to publication.

## GUARANTOR

José Martín Alanís Naranjo.
